# Bis(terpyridine) Iron(II) Functionalized Vertically-Oriented Nanostructured Silica Films: Toward Electrochromic Materials

**DOI:** 10.3389/fchem.2020.00830

**Published:** 2020-09-15

**Authors:** Neus Vilà, Alain Walcarius

**Affiliations:** Université de Lorraine, CNRS, LCPME, Nancy, France

**Keywords:** electrochromism, mesoporous silica thin films, coordination complexes, electrochemistry, optical properties

## Abstract

Recent and potential applications of electrochromic materials include smart windows, optoelectronic devices, and energy conversion. In this study, we have incorporated bis(terpyridine) iron (II) complexes into vertically-oriented silica thin films deposited on indium-tin oxide (ITO) and their electrochromic behavior has been investigated. If 2,2′:6′,2″-terpyridine is commonly used as a ligand for forming metallo-supramolecular assemblies, with the objective to get metal-terpyridine complexes with multiple stable redox states, their simple and reliable arrangement into linear structures enabling effective electronic communication is however more challenging. We propose to overcome this difficulty by generating such complexes within vertical nanochannels on electrode. Terpyridine ligands were firstly immobilized by combining a click chemistry azide/alkyne approach with an electrochemically-assisted self-assembly (EASA) method used to grow an oriented mesoporous silica membrane bearing azide groups which were further derivatized with 4′-ethynyl-terpyridine ligands. The resulting terpyridine-functionalized films were consecutively dipped in an aqueous solution of Fe(BF_4_)_2_ and then in a solution of terpyridine in acetonitrile to form the bis(terpyridine) iron (II) complexes in situ. The electrochromic properties of the films functionalized at various levels were examined by monitoring the changes in their UV/Vis spectra upon electrochemical oxidation at controlled potential of +1.2 V vs. Ag/AgCl. Due to facile charge delocalization during the Fe^2+^ to Fe^3+^ redox process, the bis(terpyridine) iron (II) functionalized silica films exhibited electrochromic properties by changing from violet to non-colored using TBABF_4_ in acetonitrile as an electrolyte. The bis(terpyridine) iron(II) film experienced reversible electrochromic switching by applying +0.5 V in a reverse reduction electrochemical process. The Fe(tpy)_2_-functionalized silica thin films displayed a good contrast ratio (ΔT%) of 47% and relatively high coloration efficiency (CE) of about 245 cm^2^/C with a response time of coloring and bleaching of a few seconds (< 4 s).

## Introduction

Electrochromic materials (ECMs) are known to undergo changes in electronic absorption bands in a reversible way because of interconversion between two or more redox states in response to externally applied potentials (Deb, 1969; Rosseinsky and Mortimer, 2001; Heuer et al., 2002; Dyer et al., 2007; Monk et al., 2007). A lot of interest has been devoted recently to electrochromic devices due to their low energy consumption as well as their memory effect (Platt, 1961; Mortimer, 1997; Grätzel, 2001; Ahn et al., 2003; Cho et al., 2005). Development of novel electrochromic materials exhibiting good durable stability, good optical contrast, coloration efficiency, and fast response time (color switching ability) at different potentials has experienced significant progress since they are useful since they are good candidates for potential applications such as information and optical storage, smart windows, and color displays (DeLongchamp and Hammond, 2001; Cutler et al., 2002; Peters and Branda, 2003; Argun et al., 2004; Wang et al., 2020). The most frequently investigated electrochromic molecules are conducting organic polymers (Groenendaal et al., 2003; Li et al., 2009; Liu et al., 2010; Amb et al., 2011) which can be eventually modified with side chain functions contributing to the enhancement of optical properties (Xiong et al., 2019) and metal oxides (Bach et al., 2002; Lee et al., 2006).

Iron (II) polypyridyl complexes are of great interest to build ECMs due to their chromophoric character due to the well-known dependence of their corresponding visible absorption bands on the oxidation state of the transition metal and exceptional stability (Han et al., 2008; Motiei et al., 2009; Dov et al., 2017; Bera et al., 2018). Previous reports have demonstrated the importance of structure in the electrochromic properties of such materials (Yoo et al., 2007; Chen et al., 2008; Higuchi et al., 2009; Wei et al., 2012; Hossain and Higuchi, 2013). In this sense, Zhong et al. have recently demonstrated that films based on oriented two-dimensional covalent organic frameworks displayed better electrochromic properties than amorphous films (Hao et al., 2019). Thus, getting ideal structures made of individual molecular wires ensuring fast electronic communication remains difficult and becomes extremely important in this context. Polychromism phenomenon induced by redox processes, and the fabrication of these materials in a convenient configuration and on different substrates are related challenges. In addition, molecular assemblies, in particular uniform thin films based on metallic complexes and their incorporation into electrochromic devices have not been extensively investigated (Higuchi, 2009; Zhong, 2013; Schott et al., 2014; Shankar et al., 2015; Banasz and Walesa-Chorab, 2019).

Mesostructured metallic materials have been obtained by chemical reduction of the metal-based precursors and the use of sacrificial templates (Jiang et al., 2018) mainly for catalytic purposes but also to generate thin films displaying interesting optoelectronic properties in which a silica template serves to preserve their optical properties (Malgras et al., 2018) and catalytic activity (Wu et al., 2006; Nandi et al., 2011). Thus, mesoporous silica thin films have potential applications in the fields of sensors, (Lee H. J., et al., 2012) catalysts, (Yu et al., 2002) optical (Frindell et al., 2002) and electrochemical applications. (Brezesinski et al., 2010) Their widely-open and regular porosity enlarges the active surface area and enhances diffusion processes through mesopores, which is interesting in particular in the case of electrochemical devices (Cheng et al., 2001; Etienne et al., 2013). In addition, the incorporation into such porous structures of guest components [e.g., dyes, (Wirnsberger et al., 2001) polymers, (Coakley and McGehee, 2003) inorganic materials, (Lee M. M., et al., 2012)] contributes to obtain new nanocomposites displaying the targeted properties. Mesoporous silica membranes with a high density of hexagonally-packed nanochannels aligned orthogonally to the underlying electrode support can be easily obtained by electrochemically-assisted self-assembly (EASA) (Walcarius et al., 2007; Vilà et al., 2014) and this method is compatible with the formation of organic-inorganic hybrid films bearing organo-functional groups covalently attached onto the internal surfaces of the silica nanochannels (Vilà et al., 2014, 2016a). When these groups are electroactive (such as ferrocene, for instance), they can be readily transformed from one redox state to another one, according to an electron transfer mechanism involving electron hopping between electroactive adjacent sites (Vilà and Walcarius, 2015) and long-range charge propagation can be maintained over the whole film thickness despite the isolating character of the silica matrix (Vilà et al., 2016b).

The fabrication of metallo-organic materials with remarkable electrochromic properties requires the deposition of uniform coatings on transparent electrodes. Among the strategies employed, the layer-by-layer techniques based on electrostatic interactions have been successfully employed by a number of groups (Li et al., 2019). In a first part of the paper we demonstrate that the versatility of the combination of EASA method and click chemistry can be extended to the introduction of polypyridyl metal-based transition complexes (M(tpy)_2_; M = Fe^2+^, Co^2+^) by using the ability of bi- or tridentate organic ligands toward coordinating transition metals. In this sense, the azide-functionalized silica thin films initially obtained by EASA are derivatized with terpyridine ligands by Huisgen cycloaddition reaction between the azide terminal groups inside the mesochannels and the 4′-(4-ethynylphenyl)-2,2′:6′,2′-terpyridine. The resulting terpyridine-functionalized films can be further derivatized taking advantage of the coordinating ability of these NNN-tridentate ligands toward transition metals. In a second part of the paper, exploiting the redox properties of Fe^2+^ ions coordinated to terpyridine ligands we explore the potential functionality of these metal-transition based complexes as electrochromic materials. Then we demonstrate that the electrochromic properties associated to the Fe(tpy)_2_ complexes are retained inside the mesochannels and strongly dependent on the functionalization degree of the films.

## Experimental Section

### Chemicals and Reagents

Tetraethoxysilane (TEOS, 98%, Alfa Aesar), (3-chloropropyl)triethoxysilane (95%, Sigma-Aldrich), ethanol (95-96%, Merck), NaNO_3_ (98%, Prolabo), HCl (37% Riedel de Haen), cetyltrimethylammonium bromide (CTAB, 99%, Acros), sodium azide (NaN_3_, 98% Sigma-Aldrich), tetrabutylammonium bromide (NBu_4_Br, 99%, Sigma-Aldrich), acetylpyridine, 4-ethynylbenzaldehyde have been used as received without further purification.

### Synthesis of 3-Azidopropyltriethoxysilane, AzPTES

AzPTES was synthesized from reaction of (3-chloropropyl)triethoxysilane (Cl-PTES) and sodium azide (NaN_3_) (Vilà et al., 2014). Cl-PTES (2.0 g, 8.3 mmol) was added to a solution of NaN_3_ (1.08 g, 16.6 mmol) and NBu_4_Br (0.644 g, 2 mmol) in dry acetonitrile. The reaction mixture was stirred under reflux for 36 h. The mixture was cooled to room temperature and the solvent evaporated under reduced pressure. The crude remaining mixture was dissolved in cyclohexane and the suspension was filtered to remove the remaining solid. Solvent was evaporated under reduced pressure at 70°C to give AzPTES as a crude oil. Yield: 1.33 g, 65%. ^1^H NMR (400 MHz, CDCl_3_): δ 0.66 (t, 2H, J = 0.85 Hz), 1.21 (t, 9H, J = 6.88 Hz), 1.66–1.73 (m, 2H), 3.25 (t, 2H, J = 7.16 Hz), m.80 (q, 6H, J = 6.88 Hz).

### Synthesis of 4′-(4-Ethynylphenyl)-2,2′:6′,2″-Terpyridine

The synthesis of 4′-(4-ethynylphenyl)-2,2′:6′,2″-terpyridine by following a procedure slightly modified from the literature (Winter et al., 2007). 2-acetylpyridine (3.63 g, 30 mmol) and 4-ethynylbenzaldehyde (1.95 g, 15 mmol) and NaOH (1.2 g, 30 mmol) were mixed and stirred for five min. Two hundred milli liter of ethanol and 100 mL of concentrated NH_3_ were subsequently added and the suspension was stirred at room temperature for three additional days. A yellow powder precipitates after these 3 days, which is filtrated and washed with water and ethanol. 1H NMR (400 MHz, CDCN_3_): 8.77 (d, J = 4.8 Hz, 2H), 8.72 (s, 2H), 8.69 (d, J = 9.0 Hz), 8.04 (t, 2H), 7.98 (d, J = 9.0 Hz, 2H), 7.70 (d, J = 9.0 Hz, 2H), 7.54 (m, 2H), 4.38 (s, 1H).

### Preparation of Azide-Functionalized Vertically-Aligned Mesoporous Silica Thin Films

The vertically-oriented silica thin films containing various amounts of azide groups have been electrochemically generated on indium-tin oxide (ITO) electrodes according to the previously reported EASA procedure using TEOS and AzPTES as silane precursors (Vilà et al., 2014). A hydroalcoholic solution (10 mL H_2_0/10 mL ethanol) containing 200 mM of the silica precursors (TEOS and AzPTES in the following ratios: 99:1, 97.5:2.5, 95:5, 92.5:7.5, 90:10, 87.5:12.5, 85: 15, 80:20, 75:25), 64 mM of CTAB as template and 0.1 M NaNO_3_ was prepared. The hydrolysis step was performed for 2.5 h upon adjusting the pH to 3 by adding 0.1 M HCl. The silica films were electrochemically generated by applying a cathodic potential of−1.3 V for 20 s [optimized values Goux et al. (2009)] to the ITO working electrode. The electrode surface was thoroughly rinsed with water and ethanol and aged overnight at 130°C. The extraction of the surfactant (CTAB) was performed by immersing the film electrode in an ethanol solution containing 0.1 M HCl for 20 min.

### Preparation of the Terpyridine-Functionalized Silica Thin Films

Preparation of terpyridine-functionalized mesoporous silica thin films was achieved via Huisgen cycloaddition between 4′-(4-ethynylphenyl)-2,2′:6′,2″-terpyridine and azide-functionalized silica films. A mixture of copper acetate (3 mg) and ascorbic acid (6 mg) dissolved in an aqueous solution (4 mL) was added to a solution of 4′-(4-ethynylphenyl)-2,2′:6′,2″-terpyridine (6.4 mg) dissolved in dimethylformamide (8 mL). The azide-functionalized silica thin film was immersed in this solution at room temperature for 34 h in the dark. After this period, the electrode was rinsed carefully with water and DMF.

### Derivatization of Terpyridine-Functionalized Silica Thin Films by Formation of the Coordination Complexes

Terpyridine-functionalized silica thin films were dipped in an aqueous solution containing [Fe(BF_4_)_2_] for 2 h. The Fe-tpy-functionalized films were subsequently dipped in a solution of acetonitrile containing 1 mM of terpyridine. The silica films turned immediately to violet confirming the formation of Fe(tpy)_2_ complexes inside the nanostructured membrane. The films were thoroughly rinsed with water and ethanol.

### Preparation of the Gel Electrolyte and Fabrication of Electrochromic Devices

The gel electrolyte was prepared by mixing LiClO_4_ (0.225 g) with propylene carbonate (1.5 mL) and acetonitrile (5.3 mL) followed by the addition of poly(methylmethacrylate) (PMMA, 0.5 g) under stirring. The mixture was stirred at room temperature for 3 h. A transparent gel was obtained and was placed on an ITO substrate (which will be used as counter electrode later on) and dried for 24 h. Another Fe(tpy)_2_-functionalized silica thin film on ITO was used as working electrode. Both ITO substrates were sandwiched to fabricate the device and an alternative voltage from +1.5 to −0.5 V was applied to measure the electrochromic properties.

### Apparatus

#### Electrochemical Measurements

Cyclic voltammetry (CV) and amperometry measurements were performed with a μ Autolab potentiostat. A one-compartment electrochemical cell with a classical three-electrode configuration was used for all the electrochemical measurements. An Ag/AgCl electrode was used as reference and the counter electrode was a platinum gauze of large surface area. The working electrode was an indium-tin oxide plate modified with the azide and/or terpyridine functionalized films. Cyclic voltammograms were recorded at 20 mV s^−1^ (unless specified otherwise, especially in the experiments concerning the effect of the scan rate on the electrochemical behavior) from 0.1 M TBABF_4_ in acetonitrile.

#### UV/Vis Spectroscopy

UV/Vis spectra were recorded on a Cary 60 spectrophotometer. The absorbance was measured by using the Cary WinUV-Scan software in a range of wavelengths between 350 and 700 nm. The transmittance measurements were performed using the Cary Win UV-Kinetics application. Blank measurements were used to compensate the background absorption and were recorded using bare ITO substrates.

#### X-Ray Photoelectron Spectroscopy

XPS measurements were carried out on ITO/glass substrate. The measurements were carried out with a Kratos AXIS ULTRA equipped with a monochromatic Al Kα X-Ray source (hν = 1486.6 eV) in an analytical chamber that was maintained at low pressure (10^−9^ mbar). All the binding energies were calibrated using the C 1 s binding energy peak centered at 284.5 eV as a reference. Curve-fitting of the XPS data has been done by using Gaussian-Lorentzian functions with CasaXPS sotware.

## Results and Discussion

**Figure 1:**
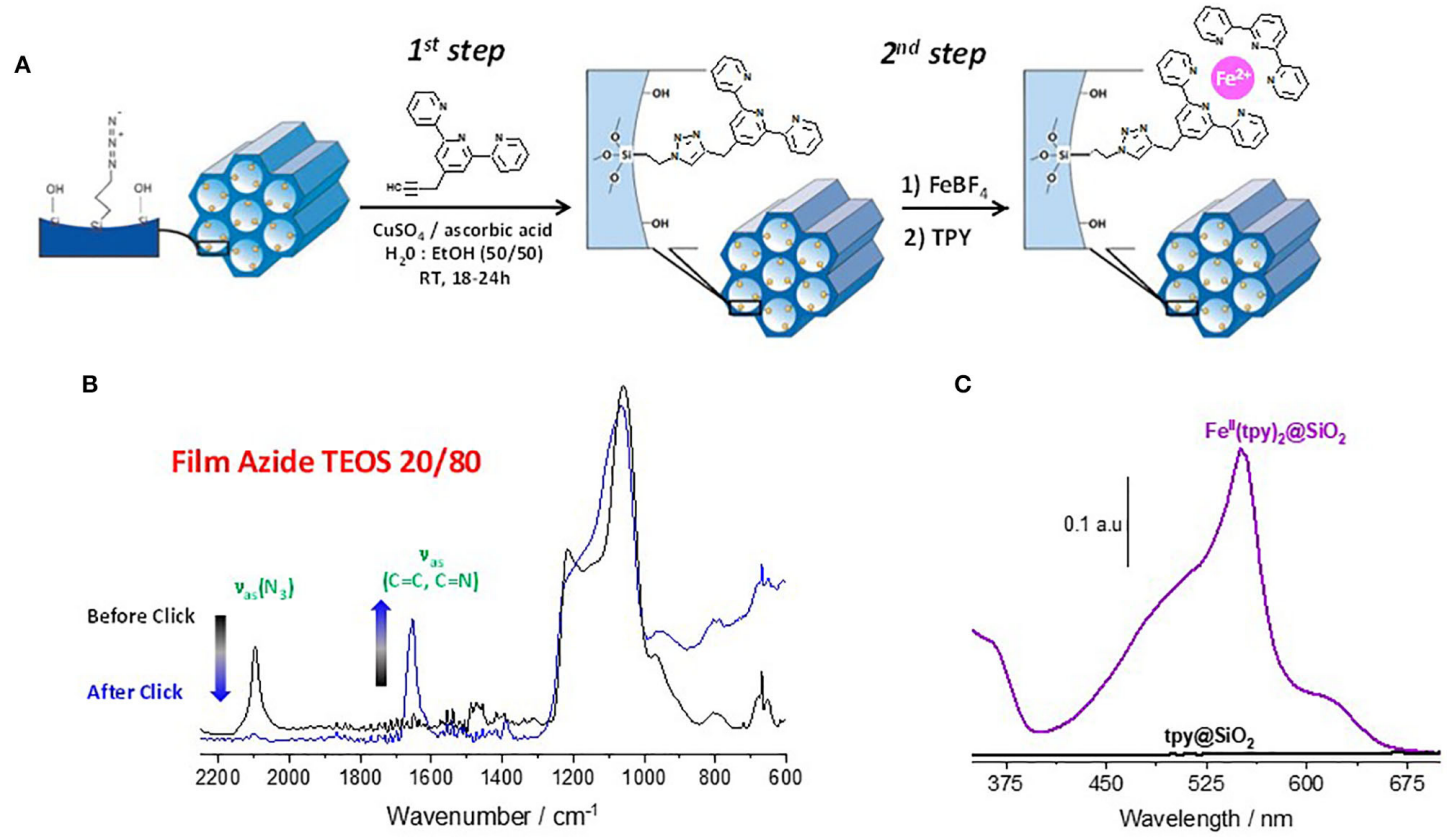
**(A)** Illustration of the fabrication process of the electrochromic molecular assemblies from azide-functionalized silica thin films. 1^st^ step: preparation of the terpyridine-functionalized silica from azide-functionalized silica films via copper-catalyzed azide-alkyne cycloaddition reaction. 2^nd^ step: Preparation of the Fe(tpy)_2_-functionalized silica thin films by coordination of the tridentate nitrogen-based ligands with Fe^2+^ and additional terpyridine ligands. **(B)** Click reaction monitored by infrared spectroscopy. **(C)** Fe(tpy)_2_ complex formation monitored by UV-Vis spectroscopy.

Terpyridine is an easily functionalizable and attractive tridentate nitrogen-based chelating ligand that has a strong coordination ability with various first-row transition-metal ions under mild conditions and with high yields. It can coordinate with d^6^ transition metal ions (e.g. Fe^2+^) giving rise to the formation of octahedral structures. The 4′-(4-ethynylphenyl)-2,2′;6′,2″-terpyridine ligand has been used here to get terpyridine-functionalized mesoporous silica films by cycloaddition Huisgen reaction starting from azide-functionalized silica films containing various amounts of azide groups (as defined from the TEOS/AzPTES molar ratios (varying from 99:1 to 75:25) in the EASA synthesis medium (Figure 1A). The success and completion of the click reaction was monitored by infrared spectroscopy taking advantage of the disappearance of the asymmetric stretching absorption characteristic of the azide moieties located at 2095 cm^−1^ (Figure 1B). Furthermore, a new strong absorption band related to the aromatic C=C stretching vibrational mode characteristic of the presence of terpyridine ligands in addition to the triazole moieties is clearly visible at 1,648 cm^−1^ confirming the successful incorporation of the terpyridine moieties (Figure 1B). These terpyridine ligands covalently attached inside the mesochannels are able to form complexes with divalent transition metal ions (Fe^2+^ for instance) after dipping the terpyridine-functionalized silica films in an aqueous solution of 0.1 M Fe(BF_4_)_2_ for 2 h, providing they are close enough to each other. Bis-complexes can be subsequently formed by dipping the intermediate Fe(tpy)-based films in a solution of terpyridine ligands in acetonitrile that will complete the coordination sphere leading to the formation of Fe(tpy)_2_ complexes in such vertically-aligned mesoporous silica thin films, which can be evidenced by UV-Vis spectroscopy (Figure 1C). The XPS data confirm the presence of Fe(II) and N. The peak positions are observed at 708 and 721 eV for Fe_2p_ and 400.1 eV for N_1s_ orbitals, respectively (Supplementary Figure 1). The iron(II) bisterpyridine complex has a linear octahedral structure with a reversible electrochromic behavior that can be readily monitored by the intensity of the metal-to-ligand charge transfer (MLCT) absorption at 555 nm, which is modulated upon electrochemical oxidation/reduction processes of the Fe^3+^/Fe^2+^ redox couple (Figure 1C).

### Characterization, Optical, and Electrochemical Properties of Fe(tpy)_2_ in Solution

**Figure 2:**
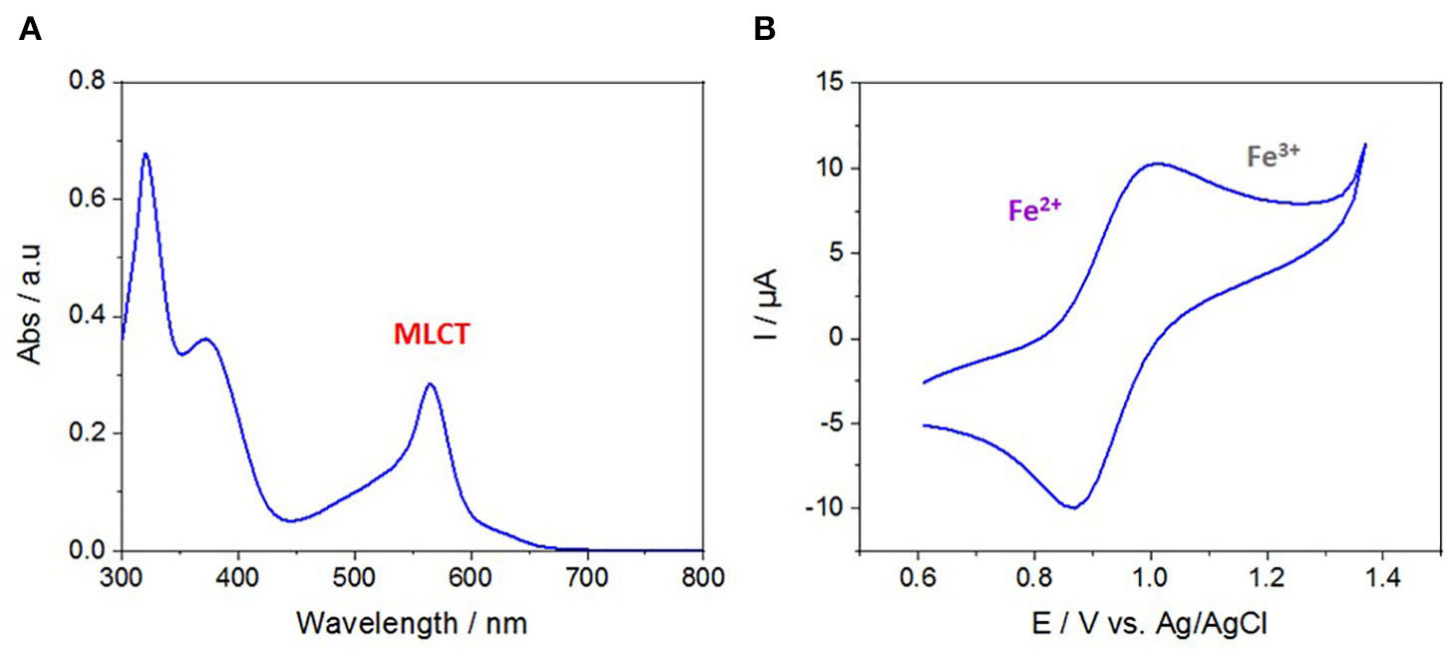
**(A)** UV-Vis absorption spectrum of a solution containing 0.5 × 10^−5^ M [Fe(tpy)_2_(BF_4_)_2_] in acetonitrile. **(B)** Cyclic voltammogram of 0.5 mM [Fe(tpy)_2_(BF_4_)_2_] in 0.1 M TBABF_4_ in acetonitrile at 50 mV s^−1^.

Before starting with the spectroscopic and electrochemical characterization of the terpyridine-functionalized mesoporous films, the optical and electrochemical properties of [Fe(tpy)_2_(BF_4_)_2_] in solution have been investigated by UV/Vis spectroscopy and CV (Figure 2). The UV/Vis spectrum showed three absorption bands: a first one located at 320 nm due to π-π^*^ transitions of the aromatic ligand, and two others at 372 and 565 nm which can be ascribed to d-π^*^ and MLCT transitions, respectively (Figure 2A). These characteristic wavelength values are similar to those of other reported polypyridine-Fe^2+^ complexes (Wirnsberger et al., 2001). The electrochemical properties of the [Fe(tpy)_2_(BF_4_)_2_] in solution were examined in acetonitrile medium (with 0.1 M TBABF_4_ as electrolyte). Figure 2B shows the typical cyclic voltammogram of a [Fe(tpy)_2_(BF_4_)_2_] complex with a characteristic reversible redox signal centered at +0.95 V vs. Ag/AgCl assignable to the Fe^3+^/Fe^2+^ couple.

### Effect of the Composition of the Starting Azide Functionalized Silica Films on the Optical Properties of the Resulting Fe(tpy)_2_-Based Mesoporous Silica Thin Films

**Figure 3:**
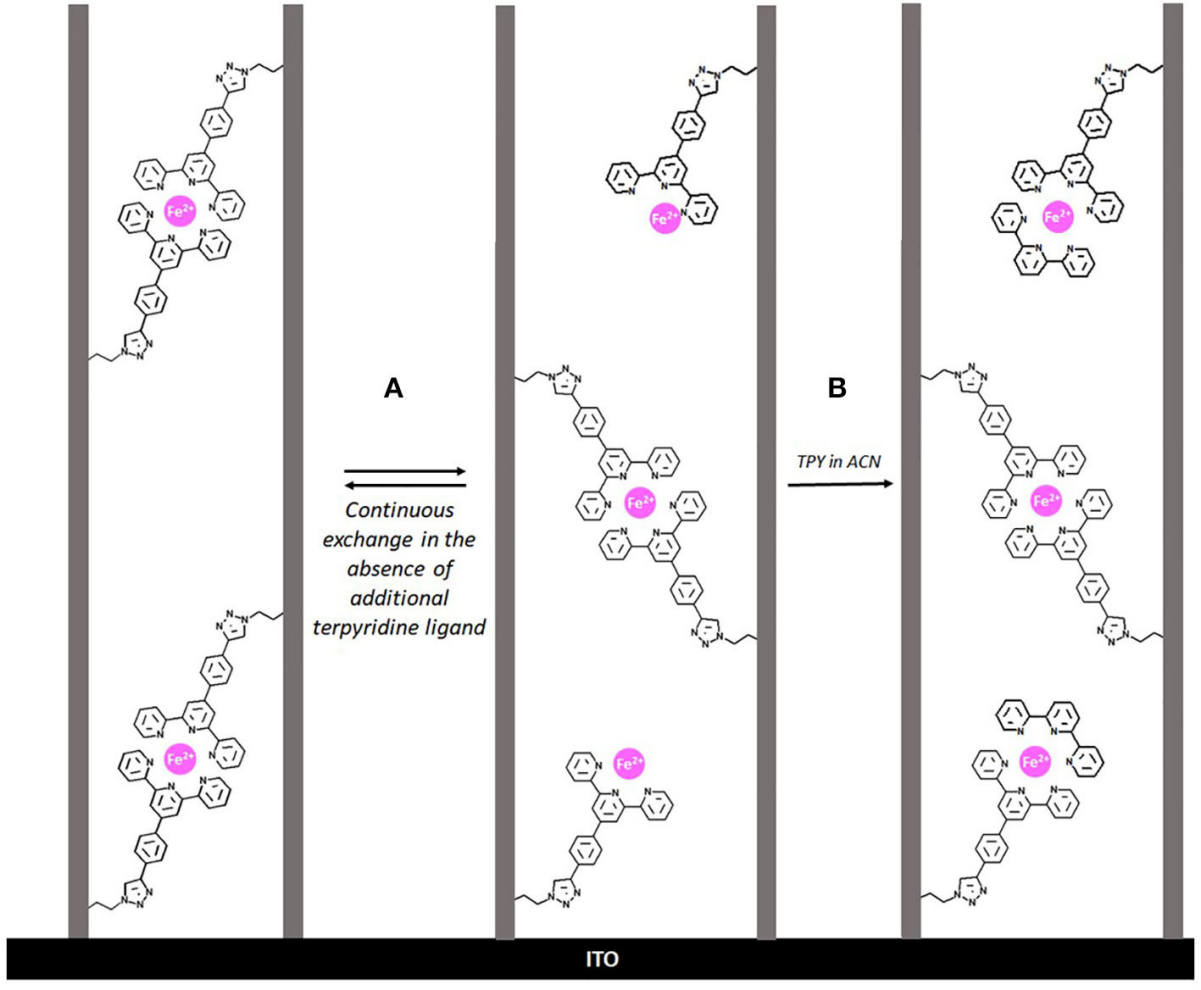
**(A)** Schematic representation of the continuous ligand exchange processes occurring inside the mesochannels in the case of the TEOS/AzPTES 90/10 starting films most likely due to the lability of the polypyridyl ligands coordinated to Fe(II). **(B)** Stabilization of the optical properties of the silica Fe(tpy)_2_-functionalized silica films stabilized by the addition of external terpyridine ligands.

The optical properties of the Fe(tpy)_2_-functionalized silica thin films can be mainly focused on the evolution of the intensity of the MLCT absorption band at 565 nm, which is responsible for the characteristic violet color of the membranes. Two important remarks have to be mentioned from preliminary observations made at the time of the preparation of the films:

1. In the case of the films prepared from TEOS/AzPTES ratio of 90/10 in the starting sol no additional terpyridine ligand is needed in the last coordination step to observe the film turning to the typical violet color characteristic of the presence of Fe(tpy)_2_. This observation indicates that the distribution of terpyridine ligands inside the mesochannels for this particular composition (in terms of the distance between the adjacent ligands and thanks to the flexibility of the linking arm) is the ideal to favor the formation of Fe(tpy)_2_ units only by dipping the terpyridine-functionalized films in the FeBF_4_ solution according to the exchange process illustrated in Figure 3 (reaction A). However, Fe(tpy)_2_-functionalized films prepared from that composition displayed quite low stability most likely due to the intrinsic lability properties of polypyridyl ligands when coordinates to first row metal transition (Hogg and Wilkins, 1962; Constable et al., 2001; Goral et al., 2001). Stabilization of the films can be achieved by addition of external terpyridine ligands (i.e., all Fe^2+^ ions coordinated by two tpy ligands, as illustrated by reaction B in Figure 3).
2. In the case of the films prepared from lowest TEOS/AzPTES ratios in the starting sol (i.e., azide-functionalized films with more than 20% of AzPTES in the starting sol), unexpectedly, we can observe with the naked eye that the Fe(tpy)_2_-functionalized films obtained are less colored compared to the ones with lower degree of functionalization. This is confirmed by a lower intensity of the MLCT absorption band at 565 nm. This is most likely due to a steric hindrance of the terpyridine ligands inside the mesochannels making more difficult the formation of the Fe(tpy)_2_ complex due to the limited space available.

**Figure 4:**
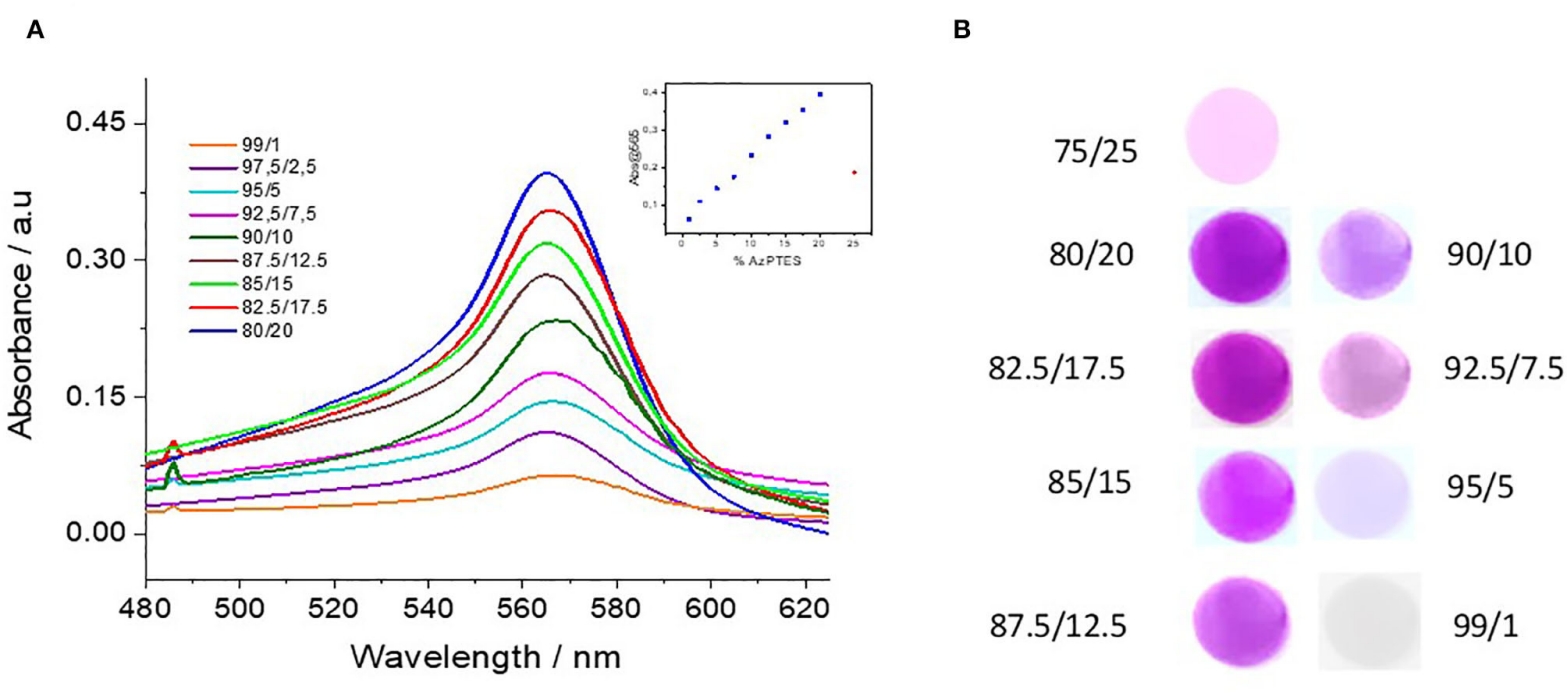
**(A)** Evolution of the spectra of the MLCT absorption band at 565 nm as a function of the % AzPTES in the starting film (insert: variation of the intensity of the absorption band at the maximum located at 565 nm). **(B)** Photographs of the Fe(tpy)_2_-functionalized silica thin films prepared from distinct TEOS/AzPTES ratios in the starting sol.

Figure 3A shows the evolution of the optical properties of mesoporous silica thin films bearing Fe(tpy)_2_ groups at various functionalization levels. Plotting of the absorption intensity of the MLCT at λ = 565 nm vs. the composition of the starting azide-functionalized silica films (insert Figure 4) corroborates a linear trend as a function of the TEOS/AzPTES ratio in the starting sol, except for the most charged film (75/25) as discussed above. This trend is consistent with the hypothesis of an effective complex formation over the whole film thickness, with amounts of complexes directly proportional to the terpyridine content in the material.

### Electrochemical Properties of the Fe(tpy)_2_-Based Mesoporous Silica Thin Films: Effect of the Functionalization Degree and the Scan Rate

**Figure 5:**
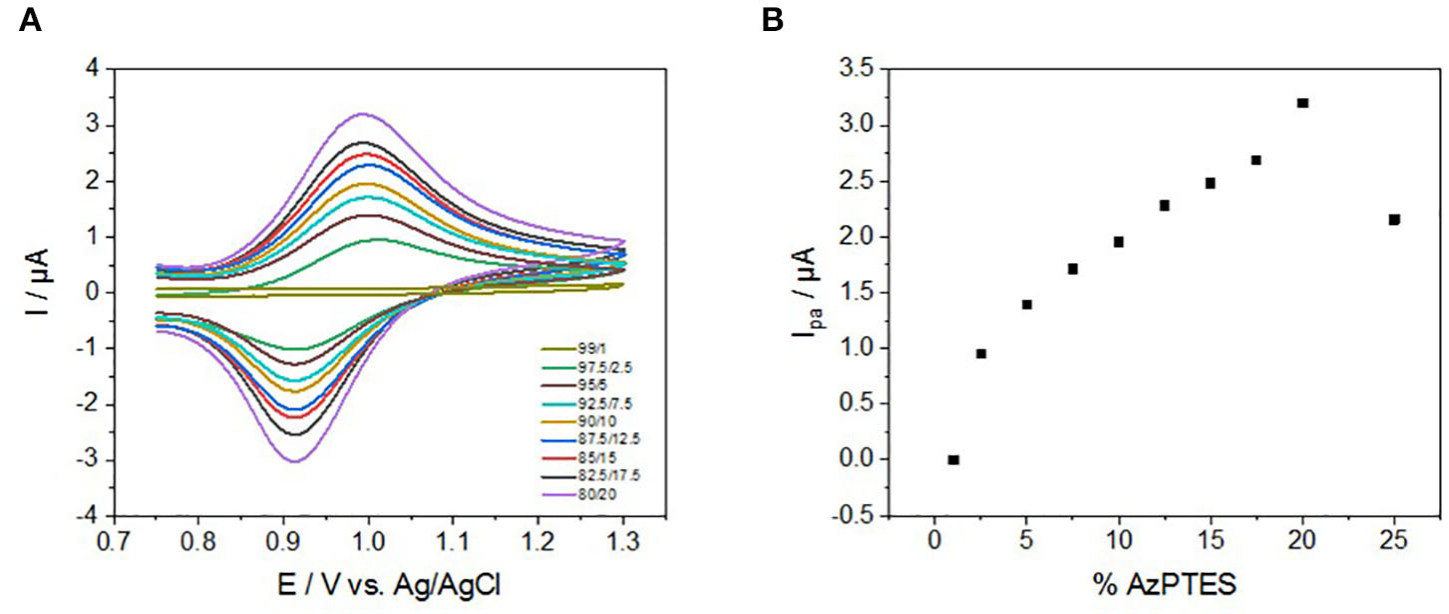
**(A)** Cyclic voltammograms of Fe(tpy)_2_-based silica films prepared from various TEOS/AzPTES ratios (total concentration of silane (TEOS + AzPTES): 200 mM) at 10 mV s^−1^ in 0.1M TBABF_4_ in acetonitrile. **(B)** Corresponding variation of the anodic peak current as a function of the % AzPTES in the starting sol.

The electrochemical behavior of Fe(tpy)_2_-functionalized silica films deposited on ITO was studied by cyclic voltammetry in acetonitrile (+0.1 M TBABF_4_ as the electrolyte) in a three-electrode system. Experiments were first carried out at a single and low potential scan rate (10 mV s^−1^) on film electrodes containing various spatial densities of the Fe(tpy)_2_ complex inside the mesochannels (composition in terms of TEOS/AzPTES ratio in the starting sol varies from 99/1 to 75/25). As illustrated in Figure 5A, cyclic voltammograms show, in all cases, a reversible signal in the potential window between +0.7 and +1.3 V vs. Ag/AgCl, in good agreement with the electrochemical behavior of Fe(tpy)_2_ in solution (Figure 2B). The CV curves are clearly related to Fe^(III)^/Fe^(II)^ redox couple, exhibiting redox processes centered at +0.95 V (with anodic-to-cathodic peak potential separation of 80 mV, yet extending up to 90 mV for the less functionalized films). The smaller separation in peak potentials for the more functionalized films suggests faster charge transfer kinetics [i.e., promoted self-exchange reaction when Fe(tpy)_2_ centers are closer to each other, as reported for other redox-active mesoporous films Vilà et al. (2015)]. Down to 2.5% AzPTES, the CV curves were still very well-defined, but at the lowest Fe(tpy)_2_ content (film prepared from 1% AzPTES) no signal can be noticed, which can be explained by a too long distance between the few Fe(tpy)_2_ moieties in the nanochannels for which no efficient electron hopping can occur (Li et al., 2012). Plotting peak currents vs. % AzPTES indicate a clear evolution related to the degree of functionalization (Figure 5B), resulting in an increase in current intensity for increasing amounts of Fe(tpy)_2_ units incorporated into the films. A charge of ca. 74 μC can be calculated from the integration of peak currents corresponding to the film prepared using 80/20 TEOS/AzPTES ratio, which corresponds to 6.2 × 10^−10^ mol Fe(tpy)_2_. This would correspond to 0.16 mmol g^−1^ of electroactive complexes in the film (calculation made on the basis of a film diameter of 8 mm, a thickness of 100 nm and the density of mesoporous silica estimated at 0.8 g cm^−3^ Edler et al., 1997. Such high concentration value suggests that huge amount of Fe(tpy)_2_ species in the film are electrochemically accessible despite the insulating character of the silica matrix, confirming the possible and effective long-range charge transfer by electron hopping through such grafted nanochannels (Vilà et al., 2016b). Electrochemical stability of the Fe(tpy)_2_-based silica thin films was investigated by multisweep cyclic voltammetry. Up to three hundred oxidation-reduction successive scans were performed between 0.5 and 1.3 V at 200 mV s^−1^ showing a loss of only 15% of the oxidation peak current.

**Figure 6:**
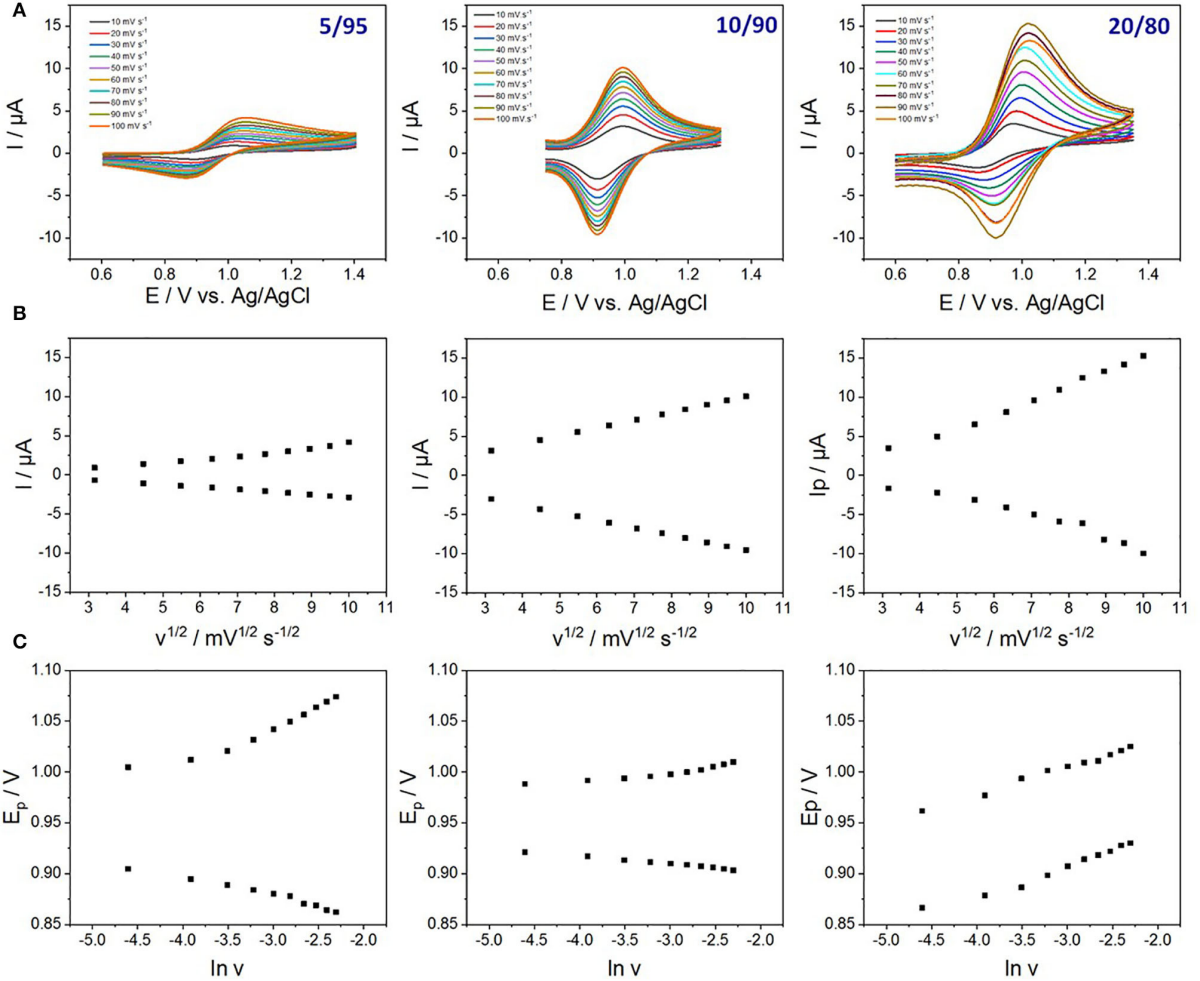
**(A)** Cyclic voltammograms of Fe(tpy)_2_-functionalized films prepared from various TEOS/AzPTES ratios (total concentration: 200 mM), as recorded at potential scan rates of 10, 20, 30, 40, 50, 60, 70, 80, 90, and 100 mV s^−1^, in an electrolyte solution containing 0.1 M TBABF_4_ in acetonitrile. **(B)** Dependence of the peak current on the scan rate (10–100 mV.s^−1^): Linear correlation between the peak current and the square root of scan rate. **(C)** Corresponding variations of peak potentials as a function of the logarithm of scan rate.

Additional information can be obtained from investigating the effect of potential scan rate on the CV response of the film electrodes containing distinct amounts of Fe(tpy)_2_ complexes (Figure 6A). In all cases, peak currents were directly proportional to the square root of potential scan rate (Figure 6B), indicating diffusion-controlled processes, at the exception of the lowest scan rates (<20 mV s^−1^) for which a contribution of thin-layer behavior can be expected (Vilà and Walcarius, 2015). Actually, the redox processes involve the electron transfer reaction itself (Fe^(III)^/Fe^(II)^) and charge balance by the electrolyte ions (i.e., ingress of anion, X^−^, to balance the excess of positive charge generated by the oxidation of Fe(tpy)_2_ in the film, Equation 1).

(1) $$\begin{array}{l} {\left[Fe{\left(tpy\right)}_{2}\right]}_{\left(\text{film}\right)}^{2+}-1e^{-}+X_{\left(\text{sol}\right)}^{-}↔{\left\{{\left[Fe{\left(tpy\right)}_{2}\right]}^{3+},X^{-}\right\}}_{\left(\text{film}\right)}\; \end{array}$$

(with tpy = terpyridine; X^−^ = anion; sol = solution)

Accordingly, the rate determining step can be either the pseudo-diffusion of the electrons (in the hopping process), the mass transport of counter-anions through the film, or a combination of both. At low functionalization levels (e.g., below 20%), the more limiting factor should be the rate of electron self-exchange between adjacent redox sites because they are more distant from each other, as sustained by larger values of anodic-to-cathodic peak separation (ΔE), especially at high potential scan rates and lowest functionalization level (Figure 6C). At higher functionalization levels (e.g., 20% and above), the electron hopping is even faster but the nanochannels containing more Fe(tpy)_2_ species leave less available free space for counter-anion diffusion, which tends to become a rate limiting factor. Such effect of counter-anion is also seen through the unusual variation of peak potentials for the reverse reaction [i.e., reduction of [Fe(tpy)_2_]^3+^, see sample 80/20 in Figure 6C] for which easier reduction is observed at high scan rate thanks to faster ejection of X^−^ from the film due to electrostatic repulsions (the silica walls are negatively charged at pH close to neutrality as experienced here in unbuffered medium), consistent with previous observations reported for ferrocene-functionalized silica thin films on electrode (Vilà and Walcarius, 2015). This sample thus appears as the most favorable one for use in experiments requiring shorter time scales (i.e., due to lower Δ*E*-values at higher scan rates), so that it will be used afterwards for electrochromic experiments.

### Electrochromic Properties of the Fe(tpy)_2_-Functionalized Vertically-Aligned Silica Thin Films

**Figure 7:**
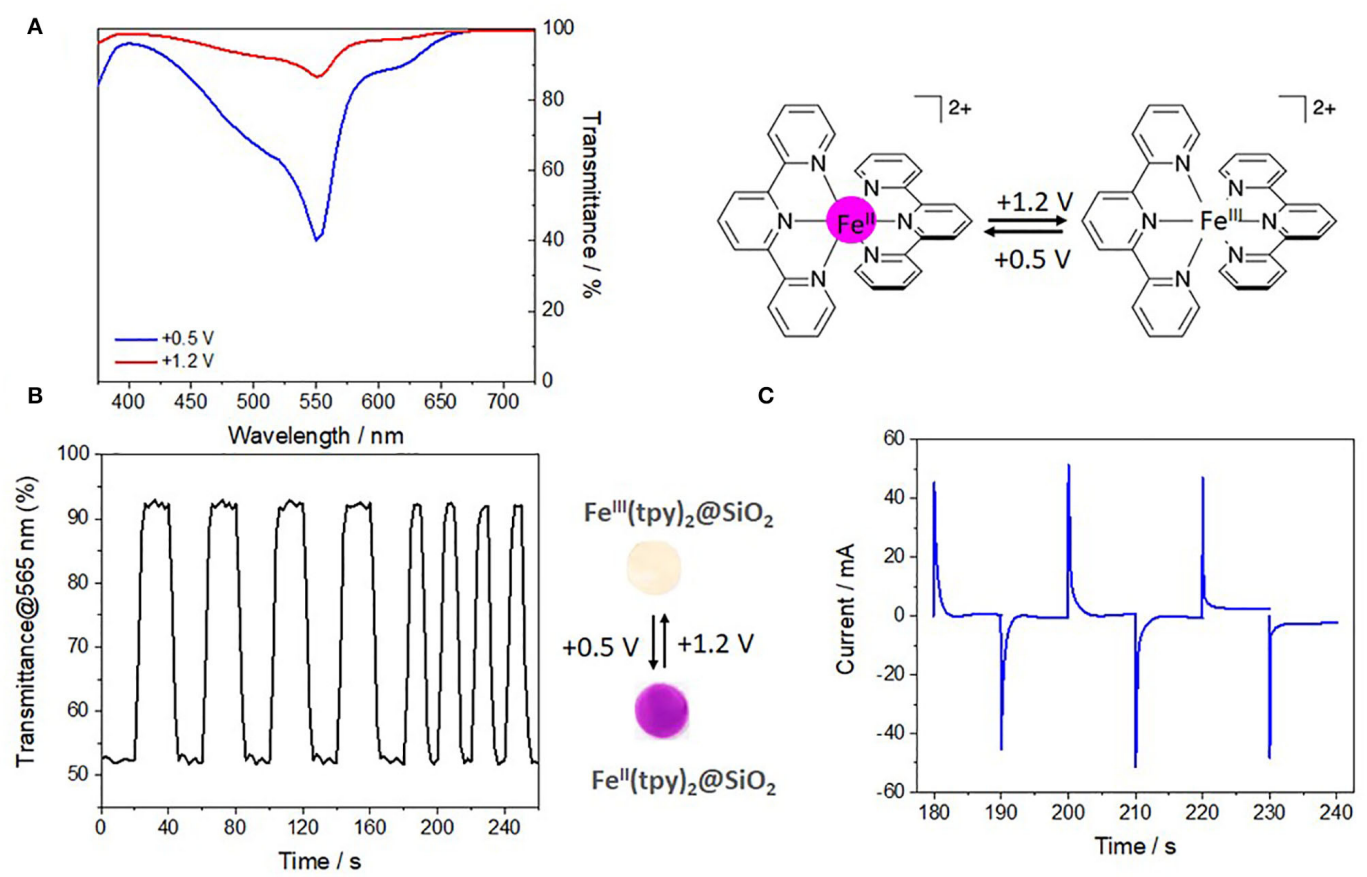
Electrochromic properties of Fe(tpy)_2_-based mesoporous silica thin film in a three-electrode system. **(A)** Transmittance spectra of the film at +0.5 V and +1.2 V. **(B)** Transmittance change at different potential pulses width of 20 and 10 s at 565 nm. Photographs of the Fe(tpy)2-functionalized silica films coated on an ITO/glass substrate in the reduced (violet) and the oxidized state (violet-shaded). **(C)** Current profile change at 565 nm of consecutive switching potential cycles of 10 s between +0.5 and +1.2 V at two different frequencies.

As pointed out above, [Fe(tpy)_2_]^2+/3+^ is likely to undergo reversible one-electron redox reactions. The metal-to-ligand charge transfer (MLCT) in the fundamental state (Fe^2+^) of these coordination complex exhibits a high molar absorptivity coefficient (ε > 2.1 × 10^4^ M cm^−1^). Oxidation of the metal center (Fe^2+^ → Fe^3+^), results in a significant decrease of the characteristic MLCT absorption band which becomes forbidden whereas a ligand-to-metal charge transfer (LMCT) grows at lower wavelength values. This is well known for the complex in solution (Hobara et al., 2007; Yoon et al., 2010) and the present work demonstrates it is also true for Fe(tpy)_2_ immobilized in the mesoporous film, via the decrease of the absorption band at 565 nm ascribed to the MLCT band of Fe^II^(tpy)_2_ originating from the disappearance of the violet color at +1.2 V (Figure 7A).

The electrochromic properties of the 20/80 Fe(tpy)_2_-based vertically-aligned mesoporous silica thin films on ITO electrode were investigated by spectroelectrochemistry in a three-electrode configuration cell by applying a double-potential step chronoamperometry (+0.5 and +1.2 V, see corresponding currents variation in Figure 7C) and in situ monitoring the change of transmittance over the time (Figure 7B). The spectroelectrochemical measurements shows a reversible change in transmittance upon oxidation-reduction of the Fe^2+/3+^ ions by applying a switching potential between +0.5 V and +1.2 V with time intervals of 20 or 10 s. The contrast ratio value defined as ΔT% of the films remains almost unchanged and close to 47% during the consecutive electrochemical cycles (up to 150 cycles were performed) suggesting robustness and good stability of the Fe(tpy)_2_-based silica thin films under applied potentials. When the potential applied increases from +0.5 to +1.2 V the color of the Fe(tpy)_2_-based silica film changed from violet to slightly violet-shaded or less intense violet (see pictures in middle bottom of Figure 6) with a simultaneous disappearance of the peak at 565 nm. The transmittance change (ΔT) at 565 nm in Fe(tpy)_2_-film was determined from the difference of the transmittances at +0.5 and +1.2 V (interval time: 10 s).

### Potential Application in the Fabrication of Electrochromic Devices

**Figure 8:**
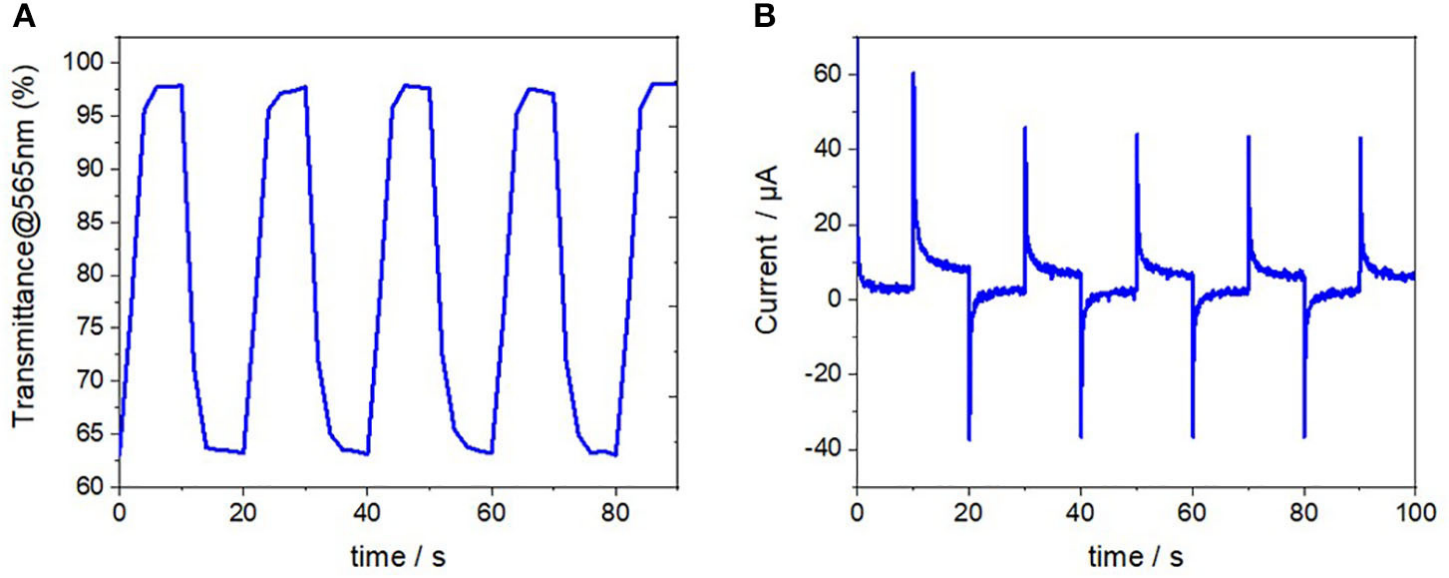
**(A)** Variation of the % transmittance over the time of the electrochromic device with a pulse width of 10s. **(B)** Current profile through the device as a function of the time at applied potentials switching from +1.5 to −0.5 V.

Preliminary tests were made to fabricate solid-state electrochromic devices using the as-prepared 80/20 Fe(tpy)_2_-functionalized vertically-oriented mesoporous silica thin films. This was achieved by using two ITO-coated glass substrates: one ITO covered with a Fe(tpy)_2_-functionalized silica film and another bare ITO plate, which were subsequently assembled to sandwich a gel electrolyte to form the solid-state electrochromic device in a configuration “glass/ITO/Fe(tpy)_2_-silica//gel//ITO/glass.” Oxidation and reduction of the coordination iron-based complex in the films are accompanied by remarkable color changes. To analyze the color changes in detail, we have performed spectroelectrochemical studies. To this aim, transmittance changes were monitored while applying consecutively alternative potentials of +1.5 to−0.5 V between the two ITO electrodes. The Fe(tpy)_2_-based electrochromic device displayed reversible color change with 43% of optical contrast (see changes in transmittance measurements over the time in Figure 8A). The stability of the Fe(tpy)_2_-based electrochromic device, exhibiting a continuous and reversible violet-to-colorless transition as a function of the applied potential, is evidenced by a constant switching current flow for at least 50 cycles (see Supplementary Figure 2). Such good stability has also to be related to the film chemical stability in the organic medium where the experiments have been performed, contrary to what could happen in aqueous media [possible degradation of the silica structure Sayen and Walcarius (2005), El Mourabit et al. (2012)]. The response times for coloration and lightening were estimated to be 2.5 and 3.5 s respectively (defined as the time taken for the 95% change of the ΔT), these values correspond well to the chronoamperometric curves (Figure 8B). Even if the response times are slightly longer than expected (2.5 and 3.5 s for coloration and bleaching processes respectively, it should be highlighted that the preliminary results obtained on the performance of such electrochromic device are comparable to the ones described in the literature especially in terms of the optical contrast (Hu et al., 2014).

## Conclusion

We have developed a versatile assembly method based on the combination of EASA and the Huisgen cycloaddition reaction with further derivatization exploiting the ability of chelating ligands to coordinate transition metal to fabricate nanostructured thin films exhibiting electrochromic properties that can be readily modulated through the applied potential. Application of these M(tpy)_2_-functionalized mesoporous silica films can open up new routes toward the generation of uniform layers with optical properties that can be modulated by charge injection and allow to reach thickness values that can vary from 25 to 400 nm which are not readily available by other dip-coating techniques. Moreover, these film electrodes can be simply configured in solid-state sandwich-type electrochromic devices composed of the Fe(tpy)_2_-based mesoporous silica film as a working electrode, an ITO plate as a counter electrode and a gel as supporting electrolyte. The Fe(tpy)_2_-based silica film showed a relatively fast, reversible, stable and robust electrochromic response with contrast ratios and coloration efficiencies that are comparable to the ones reported recently by van der Boom and co-workers in the case of the fabrication of Fe(tpy)_2_-based nanoscale coatings obtained by application of spin-coating layers of metal polypyridyl complexes (Dov et al., 2017; Lahav and van der Boom, 2018).

In this sense, the preliminary results obtained here demonstrate the feasibility of the approach consisting in the elaboration of electrochromic thin films based on isolated M(tpy)_2_ units that could be used in near future to obtain more complex nanoarchitectures based on bis(terpyridine) complexes, for example by using hexadentate nitrogen-based bridging ligands allowing the connection between metal centers of different nature. This will allow, not only the stabilization of long and continuous molecular wires based on polypyridyl ligands that are not usually stable in solution and that will be stabilized in this case by effect of their confinement in the well-organized silica matrix, but also opening the door to tune the optical properties of these nanostructures with the possibility of electronic coupling between adjacent metal centers mediated by appropriate bridge. This could generate electrochromic devices operating in the near infrared, most likely due to intervalence charge transitions. On the other hand, the use of such vertically-oriented silica films can open the door to the obtention of electrochromic layers of up to 350 nm [thanks to possible multiple EASA deposits (Giordano et al., 2017)] which is still difficult to afford by layer-by-layer techniques usually employed for the preparation of electrochromic devices.

## Data Availability Statement

All datasets presented in this study are included in the article/Supplementary Material.

## Author Contributions

NV: idea, performance of experiments, writing the manuscript. AW: writing of the manuscript. All the authors discussed the results and contributed to the final manuscript.

## Conflict of Interest

The authors declare that the research was conducted in the absence of any commercial or financial relationships that could be construed as a potential conflict of interest.
