# Supplementary material for: Bis(terpyridine) Iron(II) Functionalized Vertically-Oriented Nanostructured Silica Films: Toward Electrochromic Materials
Source: Front Chem. 2020 Sep 15;8:830. doi: 10.3389/fchem.2020.00830 (PMC7523427; doi:10.3389/fchem.2020.00830)
Supplement: Supplementary file 1 [file Data_Sheet_1.PDF]

Supporting Information

Bis(terpyridine) Iron(II) functionalized vertically-oriented nanostructured silica films: towards electrochromic materials

Neus Vilà and Alain Walcarius*

Laboratoire de Chimie Physique et Microbiologie pour les Matériaux et l'Environnement, UMR 7564, CNRS-Université de Lorraine, 405 Rue de Vandoeuvre, 54600 Villers-lès-Nancy, France

e-mail : neus.vila@univ-lorraine.fr

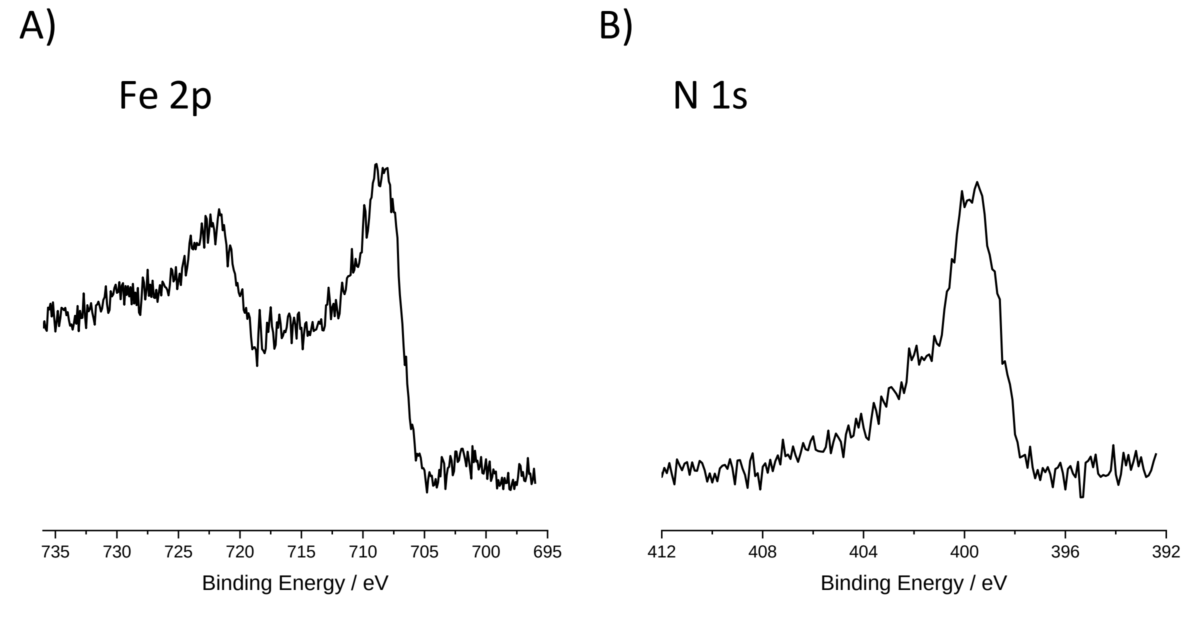

Figure S1. XPS spectra of the Fe 2p (A) and N 1s (B) regions obtained after click reaction with 4'-(4-ethynylphenyl)-2,2':6',2''-terpyridine and subsequent complexation with Fe an terpyridine ligands

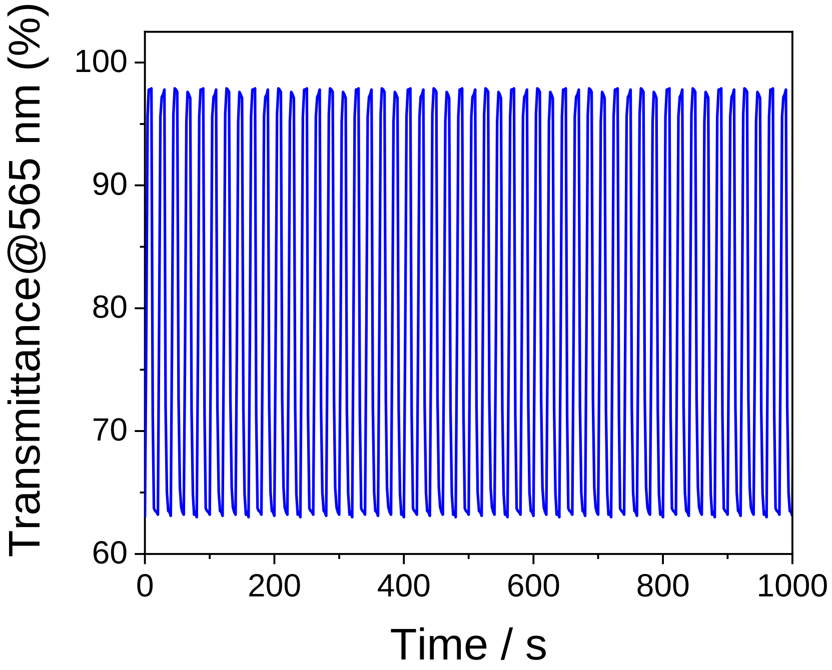

Figure S2. Variation of the % transmittance over the time of the electrochromic device with a pulse width of 20s (50 cycles).
